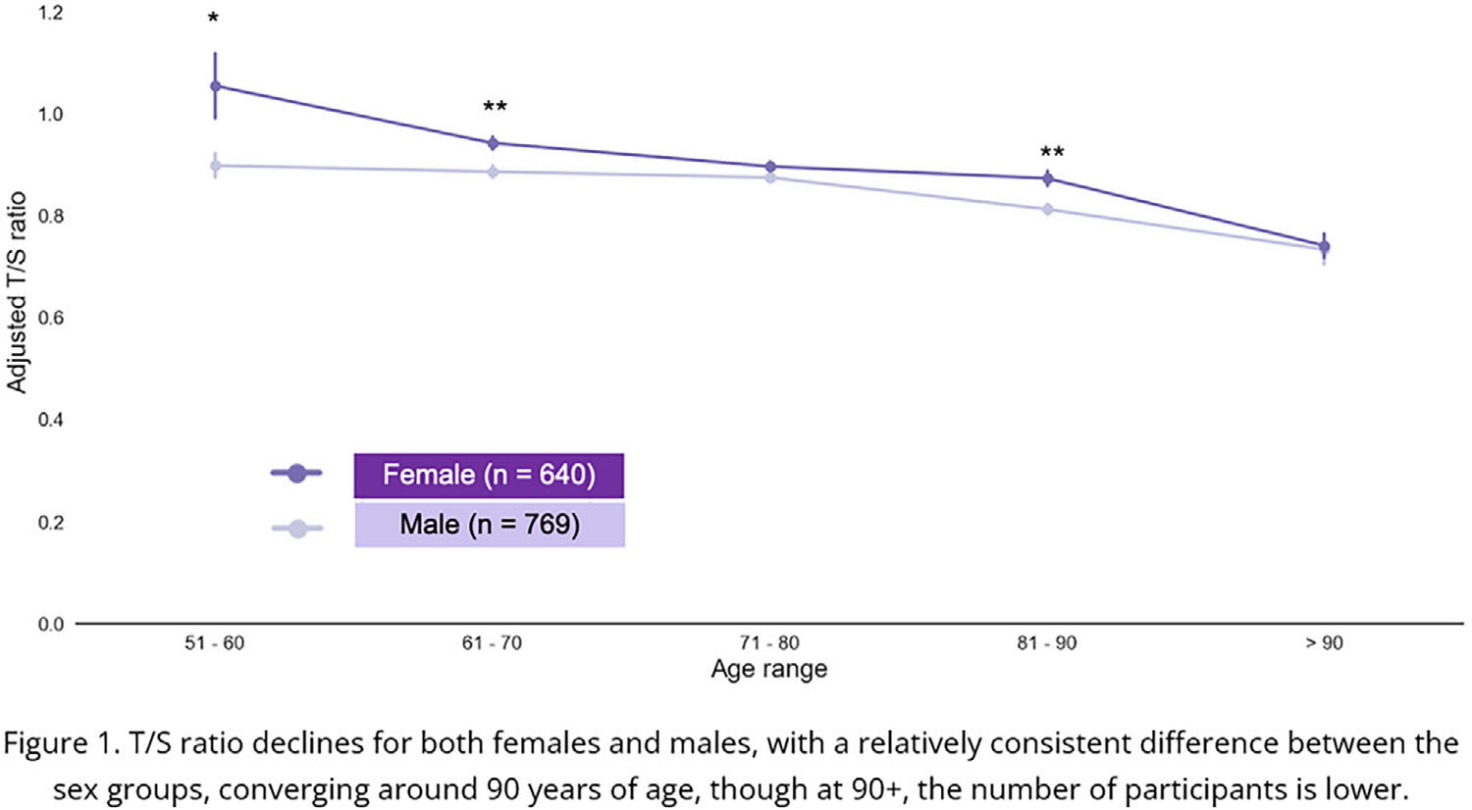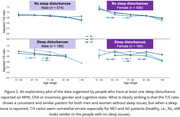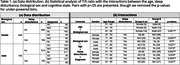# Exploring Alzheimer’s disease progression through the lens of genetics and sleep disturbances

**DOI:** 10.1002/alz.095612

**Published:** 2025-01-09

**Authors:** Leticia Fernandez‐Moguel, Ifrah Gobdon, Azizi A Seixas, Arzu Çöltekin, David Dedic, Alberto R Ramos

**Affiliations:** ^1^ University of Applied Sciences and Arts Northwestern Switzerland, Brugg‐Windisch Switzerland; ^2^ Department of Informatics and Health Science Data, University of Miami Miller School of Medicine, Miami, FL USA; ^3^ University of Miami Miller School of Medicine, Miami, FL USA

## Abstract

**Background:**

T/S ratio represents the average telomere length per genome, and has been previously linked to Alzheimer’s Disease (AD) and biological sex [1], but there is a gap in our understanding of its interactions with sleep patterns. Towards filling this gap, we present a preliminary analysis exploring correlations between *age, biological sex*, *T/S ratio* and *presence of a sleep disorder* for patients with mild cognitive impairment (MCI) and AD against a “cognitively normal” (NL) baseline.

**Method:**

Expanding on our previous work [2] we include the T/S ratio and the biological sex variables from the Alzheimer’s Disease Neuroimaging Initiative (ADNI) dataset (n = 1409). We split biological age in decades, and create a binary variable for reported sleep disturbance, i.e., No = 0, Yes = 1, in either Neuropsychiatric Inventory Questionnaire (NPIK) or Obstructive sleep apnea (OSA). We then examine correlations between the studied variables in NL/MCI/AD groups. Table 1(a) shows the sample distribution.

**Result:**

The T/S ratio decreases with increasing age, and it is statistically significantly lower for men than women on average (Figure 1, Table 1(b)), converging around age 90. In the group without sleep disturbances, no significant differences were observed for men, whereas for women, the groups (61‐70, MCI/AD) and (71‐80, NL/MCI) showed significant differences (Figure 2 & Table 1(b)). People with sleep issues exhibit erratic T/S ratio changes and more statistically significant differences depending on the cognitive state of the patient (Figure 2).

**Conclusion:**

The T/S ratio decreases with age, and is lower for males than females. Importantly, the erratic T/S ratio pattern for people with sleep issues, especially in MCI and AD groups, suggest potential new hypotheses on genetics and epigenetics. Further research and refinement of these models may contribute towards early detection and intervention strategies for Alzheimer’s disease.

**References**

[1] Nudelman, KNH, et al. “Telomere shortening in the Alzheimer’s disease neuroimaging initiative cohort.” *Journal of Alzheimer’s disease* 71.1 (2019): 33‐43.

[2] Bonfa, A, et al. “Exploring the relationship between sleep disturbances and Alzheimer’s disease using machine learning.” *Sleep* 46. Supplement_1 (2023): A370‐A370.